# In ovo technique for cell injection in the CPM followed by bead implantation in the BA2 of chicken embryos

**DOI:** 10.1016/j.mex.2020.100792

**Published:** 2020-01-14

**Authors:** Imadeldin Yahya, Denise J.M. van Lin, Marion Böing, Beate Brand-Saberi, Gabriela Morosan-Puopolo

**Affiliations:** a Institute of Anatomy, Department of Anatomy and Molecular Embryology, Ruhr University Bochum, Germany; b Department of Anatomy, Faculty of Veterinary Medicine, University of Khartoum, Sudan

**Keywords:** BA2, branchial arch, CPM, cranial paraxial mesoderm, CXCR4, C-X-C chemokine receptor type 4, DMEM, Dulbecco’s Modified Eagle Medium, HH, Hamburger and Hamilton, PBS, phosphate-buffered saline, PFA, paraformaldehyde solution, SDF-1, stromal cell-derived factor 1, Cell injection in CPM, Bead implantation in the BA2, Whole-mount immunostaining for QCPN, Chicken embryo, Quail cells, Cell injection, Bead implantation, QCPN antibody, Whole-mount immunostaining

## Abstract

The major advantage of chicken embryos model is their accessibility for microsurgical manipulations and the dissection of tissues for ex vivo explant culture. Branchial arches are embryonic structure located next to the top of developing heart. Each arch is made of surface ectoderm, endoderm, myogenic mesoderm cells and cranial neural crest cells. The myogenic mesoderm originates from cranial paraxial mesoderm (CPM), which is transiently migrated to branchial arches (BAs). The first branchial arch (BA1) mesoderm contributes to formation of mastication muscles. The second branchial arch (BA2) mesoderm gives rise to facial expression muscles. This article focuses on cell injection in the CPM and bead implantation (gain of function approaches) in the BA2. In order to follow the migration of mesoderm progenitor cells from CPM to BA2, we injected quail cells in the CPM of stage HH10-11 embryos, followed by implantation of SDF-1 bead at stage HH15-16. Later the attraction of quail cells (CXCR4^+^) towards the SDF-1 source has been observed, using whole-mount immunostaining of a specific quail antibody (QCPN) at stage HH19-22.

• Our method, which involves bead implantation followed by quail cell injection, provides useful tools for tracing migratory mesodermal cells in vivo.
• The proposed method does not require any commercial kits and can be used for various developmental process.
• It does not employ any complicated methods such as genetically engineered permanent cell labeling, multiplicity of fluorescent markers or clonal analysis.

## Highlights

• Our method involves bead implantation followed by quail cell injection and provides useful tools for tracing migratory mesodermal cells *in vivo*.
• The proposed method does not require any commercial kits and can be used for various developmental process.
• It does not employ any complicated methods such as genetically engineered permanent cell labeling or multiplicity of fluorescent markers.

## Graphical abstract

**Figure:**
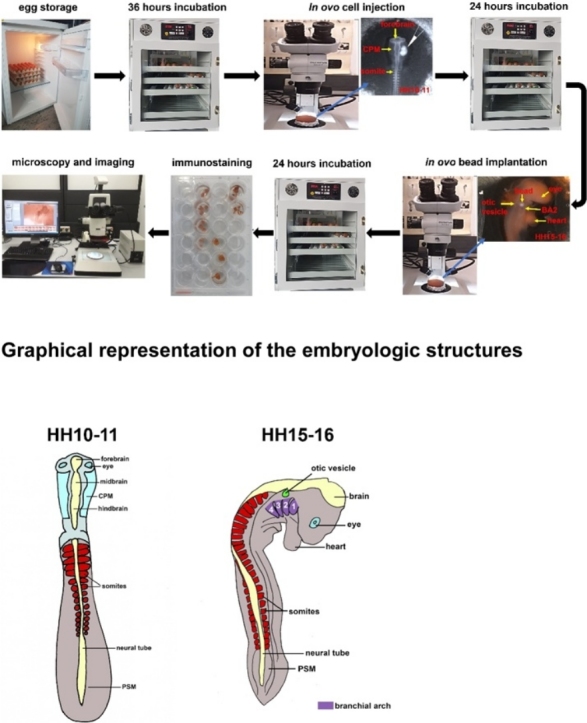

**Specification Table**

**Table:** 

Subject Area:	• *Biochemistry, Genetics and Molecular Biology* • *Medicine and Dentistry*
More specific subject area:	*Anatomy and Molecular Embryology*
Method name:	*Cell injection in CPM* *Bead implantation in the BA2* *Whole-mount immunostaining for QCPN*
Name and reference of original method:	*Kodo K, Shibata S, Miyagawa-Tomita S, Ong SG, Takahashi H, Kume T, Okano H, Matsuoka R, Yamagishi H. 2017.* *Regulation of Sema3c and the Interaction between Cardiac Neural Crest and Second Heart Field during Outflow Tract Development. Scientific reports 7.*
Resource availability:	**A. Resources needed to reproduce the experiments** *Chicken eggs* *Quail cells (QT6)* *Fridge* *Incubator* *Tungsten needle* *Forceps* *Spoon* *Medical tape* *Fine scissors* *Falcon tubes* *Pasteur pipettes* *Eppendorf pipettes* *Caps of microcentrifuge tube* *Petri dish* *24 Well plates* *Cellulose compress (900-0853, Henry Schein)* *Parafilm 38 M X 10 CM* *Aspiratory tube* *Glass capillary* *Stereo microscope (M165 FC, Leica, Germany) equipped with a digital camera (DFC420 C, Leica, Germany)* ***PBS (1x):****Start with 800 ml of distilled water, 8 g of NaCl, 0.2 g of KCl, 1.44 g of Na_2_HPO_4,_ 0.24 g of KH_2_PO_4_, adjust the Ph to 7.4 with HCl, add distilled water to total volume of 1 L.* ***PBST:****0.1% Tween-20 in 1x PBS.* ***4 % PFA in PBS:****for 1 L, add 40 g of paraformaldehyde to heated (60 °C) 800 ml of 1x PBS.* ***Blocking solution****: 2% skim milk, 1.0% Triton X-100, 1x PBST.* ***DMEM:****Dulbecco's Modified Eagle Medium* *QCPN antibody (monoclonal, 1/50, Developmental Studies Hybridoma Bank* *HRP (horseradish peroxidase) conjugated with goat anti mouse (polyclonal, 1/200, Jackson ImmunoResearch Lab, USA* *DAB (3,3′-Diaminobenzidine)* *H_2_O_2_ (30 % hydrogen peroxide solution)* *AG beads (AG 1-X2 resin, 143–1255, Bio-Rad)* *SDF-1 protein (300-28A; PeproTech)* **B. Resources needed to make video** *Olympus dual head teaching microscope* *MovieZilla Movie Maker Software* *Samsung Galaxy S8 Smartphone*

## Method details

### Chicken embryo model and egg preparation

**Fig. 1:**
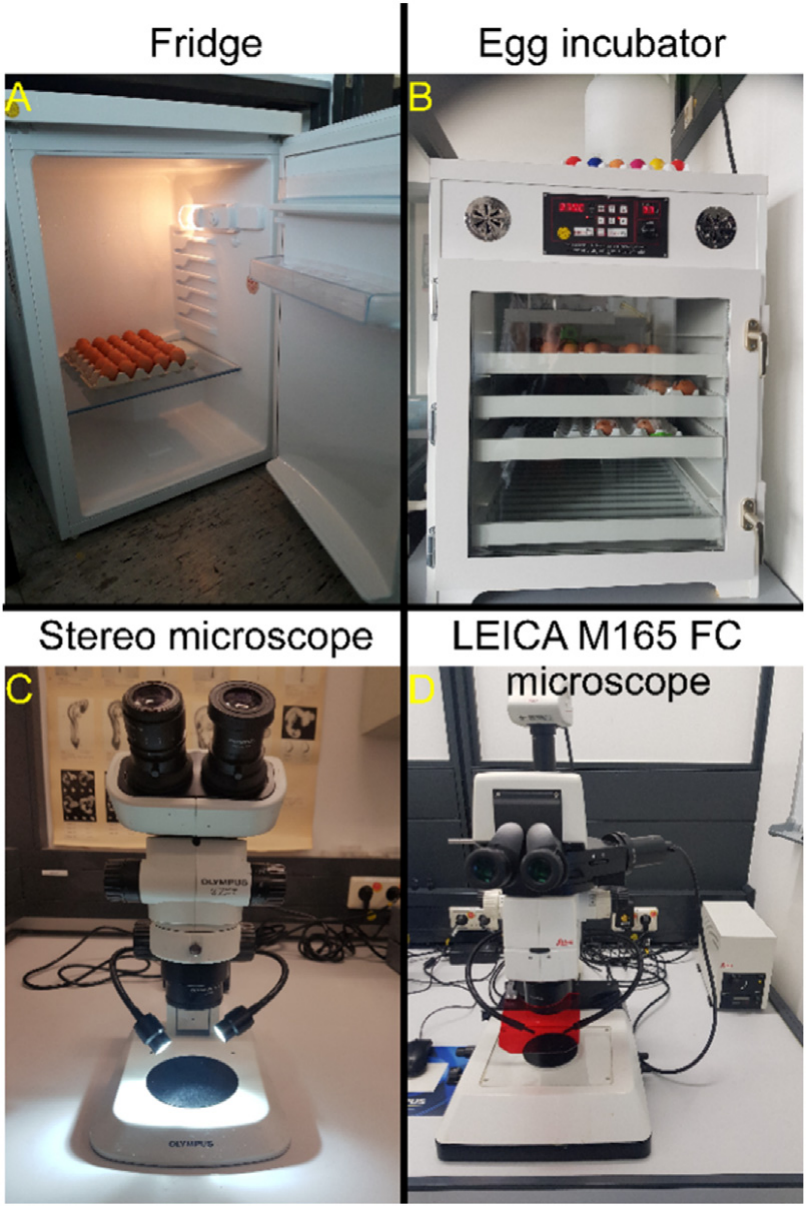
Equipment used for the preparation of chicken eggs and analysing the results throughout the experiment.

Fertilized chicken eggs (Gallus domesticus) were obtained from a local poultry breeder and stored in the fridge (Fig. 1A) at 8−16 °C. The eggs were rinsed with 70 % ethanol and incubated (Fig. 1B, J. Hemel egg incubator, Verl, Germany) at a temperature of 37 °C and at 80 % relative humidity until the stage HH10-11. At this stage, a hole was made in the side of the air chamber using small surgical scissor and 2–3 ml of albumen were withdrawn to lower the embryo using a sterile syringe (Fig. 3 and Video 1). The upper side of the egg was covered with medical tape. An oval window about 2 cm in length was opened on the same side of the egg.

### Materials necessary to make videos

**Fig. 2:**
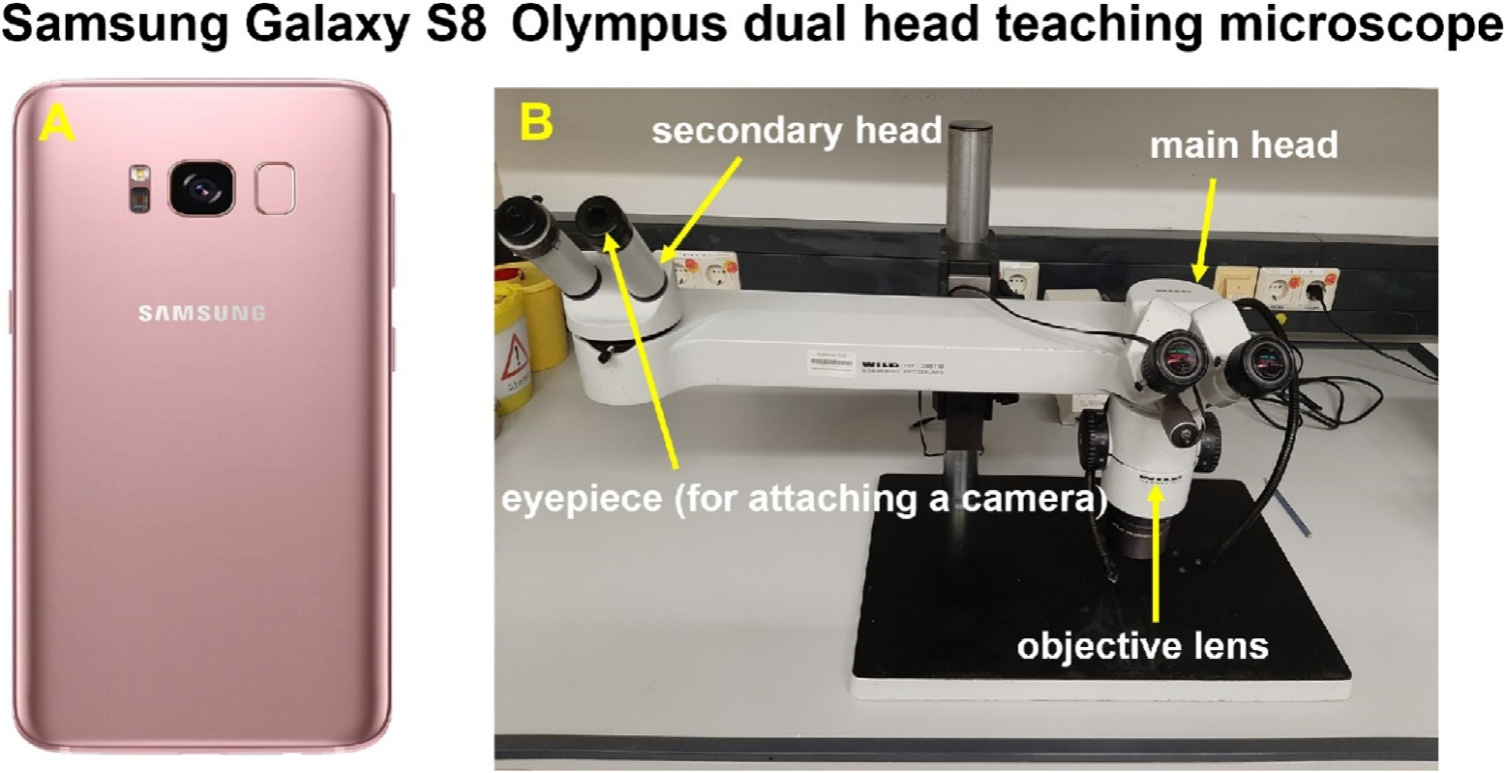
Equipment used for making video.

The Olympus teaching stereo microscope has a dual head of binocular; one is for objective lens (main head) and the other one is for digital camera (secondary head) (Fig. 2B). We used the secondary head to attach the camera of Samsung Galaxy S8 (Fig. 2A) to the eyepiece. All videos were edited using MovieZilla Movie Maker Software.

**Fig. 3:**
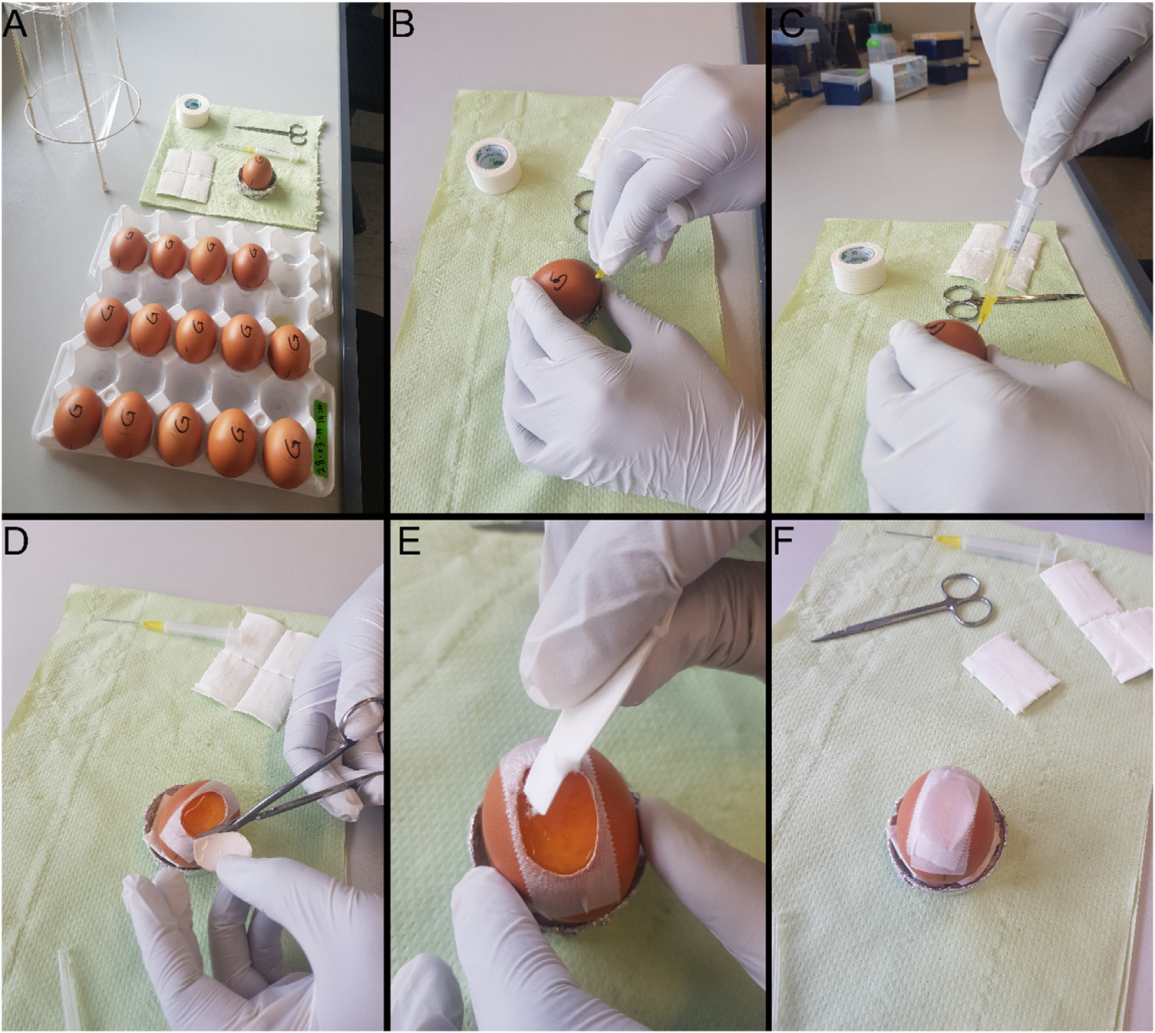
Eggs preparation.

### In ovo quail-chicken transplants

Quail cells were grown in DMEM (DMEM; Invitrogen, USA) supplemented with 10 % fetal calf serum (Invitrogen) in a 37 °C humidified atmosphere of 5 % CO_2_ in air. Cells were seeded on the day of the injection at a density of 3 × 10^5^ cells per T25 flask. Cells were harvested, centrifuged in DMEM onto microcentrifuge tube. The eggs were windowed and prepared for cell injection following the procedures previously described above. India ink diluted 1:10 in Locke’s solution containing penicillin G (Penicillin G sodium salt, PENNA, Sigma), was injected into the yolk under the blastoderm to allow visualization of the embryo. The extra membrane overlaying the embryo (vitelline membrane) was partially removed with a tungsten needle. The cell injection technique continuously used chick embryos as hosts for quail cells. The cells were injected in the cranial paraxial mesoderm of HH10-11 chicken hosts using a capillary glass needle (Video 2 and Fig. 5A). The operated eggs were sealed with medical tape and re-incubated for 24 h (HH15-16).

### Preparation of SDF-1 beads

**Fig. 4:**
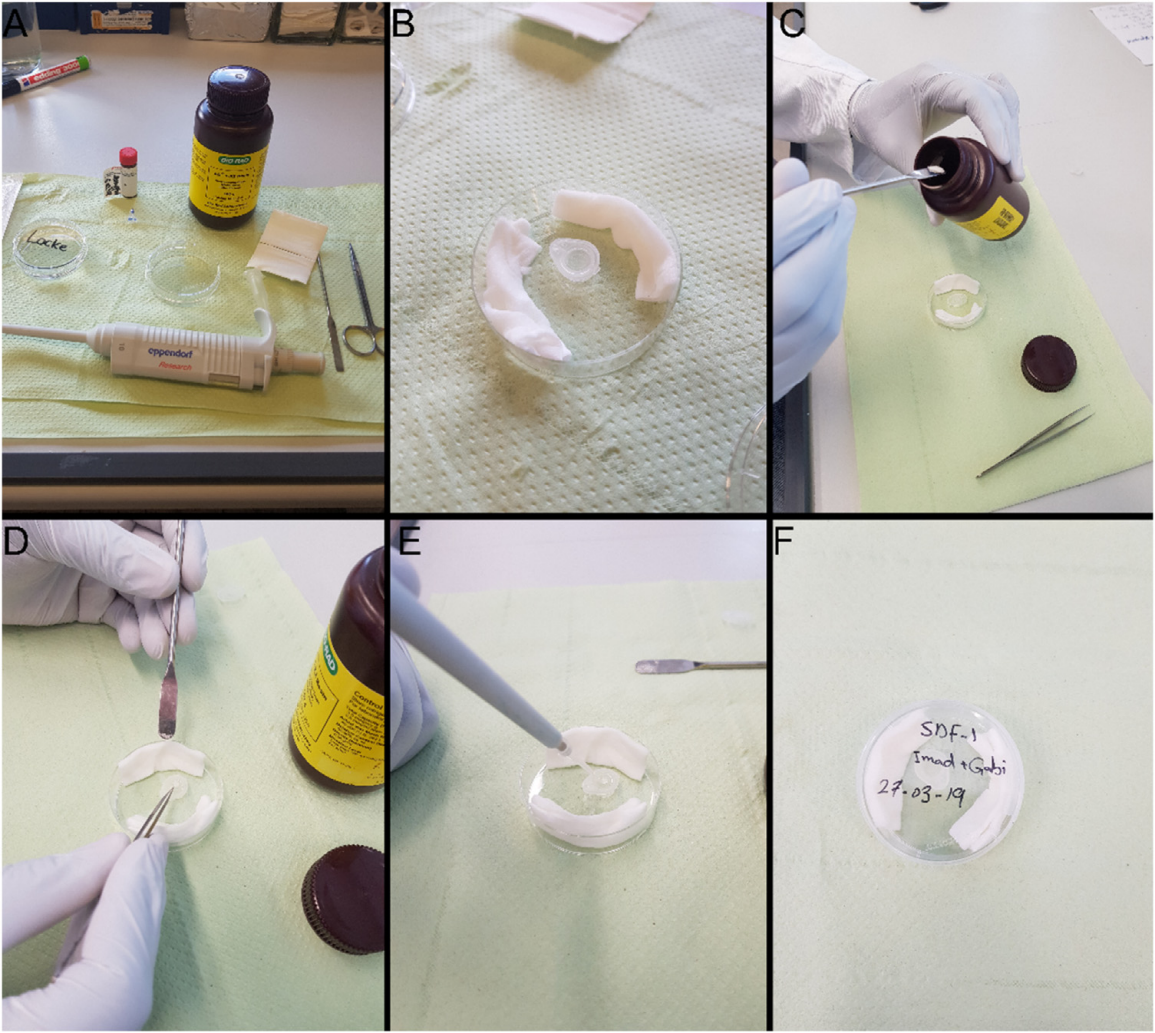
Beads preparation.

The AG beads (AG 1-X2 resin, 143-1255, Bio-Rad) were placed on the top of the microcentrifuge cap and soaked in a 1 μg/ml SDF-1 (300-28A; PeproTech). Control beads were soaked in PBS. The caps with beads were afterwards placed into small Petri dish surrounded by Cellulose compress (900-0853, Henry Schein). Few drops of Locke’s solution were adsorbed onto Cellulose compress, to create a humid environment which will avoid the dryness of the beads. The dishes were sealed with parafilm and kept at 4 °C overnight (Fig. 4 and Video 3).

**Fig. 5:**
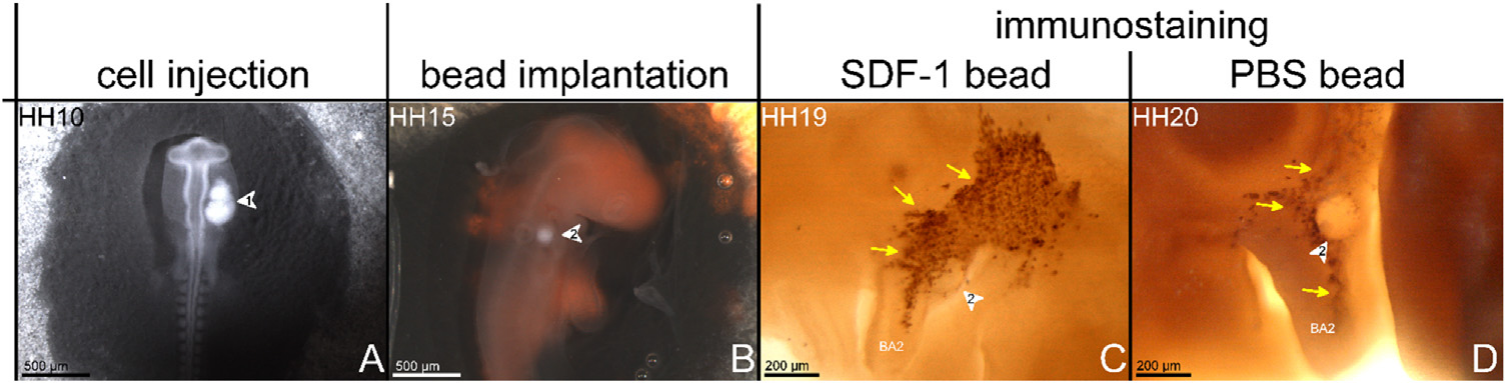
**In ovo quail cell injection, bead implantation in BA2 and whole mount immunostaining.** Stages of development are indicated at the top of the panel. (A) Dorsal view of stage HH10 embryos injected with quail cells (arrowhead 1). (B) PBS or SDF-1 beads implantation into the BA2. Arrowheads 2 indicate the beads. (C) Second branchial arch region of chicken embryos injected with quail cells. SDF-1 bead attracts QCPN positive (quail) cells (C), whereas QCPN positive cells in the PBS beads treated embryos migrated normally and located in the central portion of the BA2 (yellow arrows in photo D).

### In ovo bead implantation

When the injected embryos reached stage HH15-16, the amnion membrane overlaying the embryos was carefully removed with a tungsten needle. An incision in the BA2 tissue was created with a fine tungsten needle. Using fine forceps, the beads were inserted into the incision. PBS beads were implanted as a control. The operated eggs were sealed with medical tape and re-incubated until the stage HH19-22 (Video 4 and Fig. 5B). Finally, the embryos were fixed in 4 % PFA in PBS and processed for whole mount immunostaining.

### Whole mount immunostaining

The whole mount immunostaining method presented here is based on the method by Kodo K. et al.[1]. Embryos ranging from stage HH19 to HH22 were isolated and fixed overnight in PFA 4 % in PBS at 4 °C. The fixed embryos were dehydrated in a series of different graded of methanol in PBS at room temperature and were subsequently stored at −20 °C. Embryos were then incubated in 6 % H_2_O_2_ in methanol for 6 h at room temperature to block the endogenous peroxidase activity. The embryos were then rehydrated and blocked in blocking solution (PBST, 2 % non-fat milk, 1.0 % Triton X-100) for 1−2 h at room temperature. To detect quail cells, embryos were incubated with the primary antibody (mouse anti-quail QCPN monoclonal antibody (from Developmental Studies Hybridoma Bank, the university of Iowa)), which is specific to quail cells (Fig. 5C, D), in blocking solution (diluted 1:50) for 2 days at 4 °C. After five times washes (1 h for each wash) in blocking solution at 4 °C, the embryos were incubated with HRP (horseradish peroxidase) conjugated with the secondary antibody (goat anti-mouse (polyclonal, 1:200, Jackson ImmunoResearch Lab, USA)) in blocking solution at 4 °C overnight. The following day, embryos were washed extensively 5 times (1 h for each wash) in blocking solution at 4 °C. The staining was performed by incubating embryos in the staining solution (250 μl DAB (0.16 mg/ml), 1750 μl PBS and 2 μl H_2_O_2_ 30 %) for 15 min at room temperature for color reaction to develop. After removing the staining solution, the embryos were rinsed with PBS for 10 min at room temperature followed by the fixation overnight in 4 % PFA in PBS at 4 °C. Images were taken using a stereo microscope (M165 FC, Leica, Germany) equipped with a digital camera (DFC420 C, Leica, Germany) (Fig. 5).

## Declaration of Competing Interest

The authors declare that there are no conflicts of interest.
