# Supplementary material for: In ovo technique for cell injection in the CPM followed by bead implantation in the BA2 of chicken embryos
Source: MethodsX. 2020 Jan 14;7:100792. doi: 10.1016/j.mex.2020.100792 (PMC6994716; doi:10.1016/j.mex.2020.100792)
Supplement: Supplementary file 1 [file mmc1.docx]

**Supplementary material and/or Additional information:**

*Video 1*

*Video 2*

*Video 3*

*Video 4*
